# Dilation of Pregnant Rat Uterine Arteries with Phenols from Extra Virgin Olive Oil Is Endothelium-Dependent and Involves Calcium and Potassium Channels

**DOI:** 10.3390/cells13070619

**Published:** 2024-04-02

**Authors:** Milena Esposito, Mariacarmela Gatto, Marilyn J. Cipolla, Ira M. Bernstein, Maurizio Mandalà

**Affiliations:** 1 Department of Biology, Ecology & Earth Science, University of Calabria, 87036 Arcavacata di Rende, CS, Italy; milenaesposito17@gmail.com (M.E.); mariacarmelagatto91@hotmail.it (M.G.); 2 Department of Obstetrics, Gynecology and Reproductive Sciences, University of Vermont, Larner College of Medicine, Burlington, VT 05405, USA; marilyn.cipolla@med.uvm.edu; 3 Department of Neurological Sciences, University of Vermont, Larner College of Medicine, Burlington, VT 05405, USA; ira.bernstein@uvmhealth.org

**Keywords:** pregnancy, endothelial cells, blood vessels, smooth muscle cells, ion channels

## Abstract

During pregnancy, uterine vasculature undergoes significant circumferential growth to increase uterine blood flow, vital for the growing feto-placental unit. However, this process is often compromised in conditions like maternal high blood pressure, particularly in preeclampsia (PE), leading to fetal growth impairment. Currently, there is no cure for PE, partly due to the adverse effects of anti-hypertensive drugs on maternal and fetal health. This study aimed to investigate the vasodilator effect of extra virgin olive oil (EVOO) phenols on the reproductive vasculature, potentially benefiting both mother and fetus. Isolated uterine arteries (UAs) from pregnant rats were tested with EVOO phenols in a pressurized myograph. To elucidate the underlying mechanisms, additional experiments were conducted with specific inhibitors: L-NAME/L-NNA (10^−4^ M) for nitric oxide synthases, ODQ (10^−5^ M) for guanylate cyclase, Verapamil (10^−5^ M) for the L-type calcium channel, Ryanodine (10^−5^ M) + 2-APB (3 × 10^−5^ M) for ryanodine and the inositol triphosphate receptors, respectively, and Paxilline (10^−5^ M) for the large-conductance calcium-activated potassium channel. The results indicated that EVOO-phenols activate Ca^2+^ signaling pathways, generating nitric oxide, inducing vasodilation via cGMP and BKCa^2+^ signals in smooth muscle cells. This study suggests the potential use of EVOO phenols to prevent utero-placental blood flow restriction, offering a promising avenue for managing PE.

## 1. Introduction

During pregnancy, profound hemodynamic changes occur to accommodate the increased metabolic demands of both the mother and fetus, ensuring adequate fetal growth and development. These changes include a 40% increase in plasma volume [1], approximately a 10% decrease in blood pressure (BP) [2], and a threefold reduction in total peripheral vascular resistance compared to a non-pregnant state [3]. These alterations are primarily associated with vascular remodeling [4]. Specifically, the maternal uterine vasculature undergoes significant circumferential growth [5], resulting in a substantial increase in uteroplacental blood flow, which, at term, can increase several-fold above that in the non-pregnant state. In human females, this increase can range from 10 to 20 fold (from 50 to 700–800 mL/min), [6] and is even greater in animals such as rodents or sheep [7,8].

Failure of these physiological adaptations occurs when hypertension complicates pregnancy, particularly in the form of preeclampsia (PE). Gestational hypertension, PE, and eclampsia are classified among gestational hypertensive disorders. PE typically arises after the 20th week of pregnancy and is characterized by an elevated blood pressure (hypertension) with or without proteinuria (protein in the urine) alongside signs of damage to other organ system, such as the kidneys or liver. Furthermore, PE can progress to eclampsia, a severe complication marked by the development of seizures or convulsions. Gestational hypertension, on the other hand, is defined as hypertension developing after the 20th week of gestation without the systemic features characteristic of PE [9], and is recognized as the most common cause of perinatal mortality and maternal deaths worldwide [10,11]. Its onset is associated with a reduced uteroplacental blood flow, impeding the delivery of oxygen and nutrients to the developing fetus, thus leading to intrauterine growth restriction (IUGR) [12,13,14]. Furthermore, it has an impact on maternal health not only during gestation, but also post-delivery. Among the gestational hypertensive disorders, preeclampsia (PE), a major complication of pregnancy, has a global incidence of 4.6% [15], resulting in over 70,000 maternal deaths and 500,000 fetal deaths worldwide annually [12].

PE is characterized by inadequate vascular remodeling, increased vascular resistance, heightened sensitivity to vasoconstrictive agents, and endothelial dysfunction [16]. Despite efforts, besides labor induction, preventive or curative treatments remain elusive [17]. Managing pregnancy-related hypertensive disorders poses a significant challenge in clinical practice due to the potential adverse effects of some antihypertensive medications on fetal health. These risks include fetal growth restriction, decreased uteroplacental blood flow, neonatal hypoglicemia, electrolyte disorders, neonatal renal failure, cardiac defects, and fetopathy [18].

In this context, recent research has unveiled intriguing findings. Both in vivo and ex vivo studies, involving humans [19,20,21,22,23,24] and animals [25,26], have demonstrated the beneficial biological effects of extra virgin olive oil (EVOO), particularly its phenolic fraction on vascular function. These studies highlight EVOO’s capacity to reduce blood pressure and promote vessel relaxation [26].

In this study, we investigated the effects of the phenolic fraction of EVOO on UAs obtained from pregnant rats, marking the first exploration of such action. Our aim was to assess the vasodilatory properties of EVOO phenols on UAs, thus providing insights into the molecular pathway involved in their mechanism of action. Given their vasorelaxant effect, EVOO phenols hold potential for addressing the reduced uteroplacental flow characteristic of pregnancy-related hypertensive disorders and could be considered for the treatment of PE.

## 2. Materials and Methods

### 2.1. Extra Virgin Olive Oil Phenols

The phenolic extract utilized in this study was obtained from crude EVOO. We employed the liquid/liquid partitioning method using methanol/water (80:20) [27], a solvent combination known for its efficacy in phenolic compound extraction [27]. This solvent mixture is also specified in the official method for phenolic compound determination. The extraction process involved the use of an UltraTurrax dispersing device (IKA^®^-Werke GmbH & Co. KG, Staufen, Germany), operating at 6000 rpm for 2 min. The resulting emulsion underwent centrifugation at 4000 rpm for 15 min at room temperature. The hydroalcoholic fraction was then evaporated and subsequently reconstituted using acetonitrile to dissolve residues, with hexane employed to remove minor lipid components. Qualitative and quantitative analyses of the phenolic compounds were carried out using high-performance liquid chromatography with UV detection (HPLC-UV) [28]. Oleuropein was used as the standard to dose the fraction of the total phenols isolated from the EVOO, because it is the most abundant phenol in EVOO.

### 2.2. Animals

Female Sprague-Dawley rats from Envigo (Udine, Italy) were utilized for the experimental procedures. Pregnant rats aged 12–15 weeks were obtained by mating a female in estrus with a fertile male overnight; confirmation of day 1 of pregnancy was established by detecting spermatozoa using a vaginal smear the following morning. The rats were housed at the University of Calabria under controlled conditions, maintaining a 12 h light/dark cycle, with ad libitum access to commercial chow and tap water. All experimental protocols were conducted in accordance with the European Guidelines for the care and use of laboratory animals and were approved by the Italian Institutional Animal Care (Approval number: 530/2021-PR). Animals designated for experimental purposes were euthanized with isoflurane inhalation followed by cervical transection.

### 2.3. Rat Isolated Radial Uterine Artery Preparation

Pregnant animals in mid-pregnancy (on the 14th day of a 22 day gestation period) were utilized for experimentation. Subsequent to euthanasia, the uterus and its vasculature were carefully excised and transferred to a petri dish filled with cold (4 °C) HEPES physiological saline solution (HEPES-PSS). The radial uterine arteries (UAs) were meticulously dissected to preserve their integrity and minimize damage, employing fine forceps (Dumont #55) and Vannas spring scissors obtained from Fine Science Tools (FST, Foster City, CA, USA). Following isolation, the UAs were sectioned into segments approximately 3–4 mm in length before being cannulated onto the two cannulae of a myograph chamber [Instrumentation and Model Facility at the University of Vermont (Burlington, VT, USA)] for subsequent functional studies. Both the cannulae and the chamber were filled with HEPES-PSS. One end of the vessel was firmly attached to a cannula using nylon threads and gently perfused with HEPES-PSS to evacuate blood from the lumen. Subsequently, the other end was affixed to the remaining cannula to allow for experimentation under transmural pressures without flow. The vessels were pressurized to 50 mmHg, a pressure level that closely mimics in vivo conditions, utilizing a pressure-servo system (Living Systems Instrumentation, Burlington, VT, USA). Continuous recording of the lumen diameter was facilitated by a data acquisition software provided by Ionoptix (Westwood, MA, USA). Visualization of the vessel was achieved through a microscope connected to a TV camera, allowing for real-time monitoring. The video output signal of a selected scan line was processed by an electronic dimension analyzing system. Detailed protocols for vessel cannulation, pressurization, and lumen diameter recording have been previously published [29].

### 2.4. Experimental Protocol

The viability and endothelial integrity of the UAs were assessed by exposing the vessels to a high-potassium (80 mM, HK) depolarizing physiological saline solution (PSS) to induce contraction and acetylcholine (Ach) to induce relaxation. UAs that did not elicit reproducible responses to either HK or Ach were excluded from the study. Subsequently, the UAs were equilibrated for 30 min in HEPES-PSS at 37 °C and pre-constricted with phenylephrine to achieve a 40–50% reduction in lumen diameter [30]. Following pre-constriction, the UAs were exposed to increasing concentrations (ranging from 10^−7^ to 3 × 10^−6^ M) of EVOO phenols or its vehicle, ethanol, with changes in diameter recorded once stabilized at each concentration. Ethanol was tested in quantities corresponding to the concentrations of the EVOO phenols tested. At the end of each experiment, the vessels were treated with a relaxing solution, HEPES-PSS without Ca^2+^ containing the phosphodiesterase inhibitor papaverine (10^−4^ M), to induce maximal vasodilation. The lumen diameters and wall thickness were recorded throughout the experiment.

To investigate the potential effects of EVOO phenols on endothelial function, a series of experiments were conducted on arteries with the endothelium removed. The procedure involved cannulating the arteries at one end within a chamber and perfusing them initially with air for 10 min, followed by bi-distilled water for 5 min. Subsequently, HEPES-PSS solution was introduced to flush out the bi-distilled water from the vessel. The efficacy of this process was assessed by observing the relaxation response to acetylcholine (10^−5^ M). UAs exhibiting over an 85% relaxation response to Ach (percent reversal of phenylephrine-induced contraction) were classified as having an intact endothelium. Conversely, UAs exhibiting less than 10% relaxation were deemed without endothelium (denuded artery).

To elucidate the molecular mechanism(s) underlying the vasodilatory effects of EVOO phenols, a dose-response curve was investigated on the UAs which, prior to exposure to phenylephrine, were pre-incubated for 20 min with one of the following inhibitors: (1) Nω-nitro-L-arginine methyl ester (L-NAME, 10^−4^ M) and Nω-nitro-l-arginine (LNNA, 10^−4^ M): inhibitors of the enzyme nitric oxide synthase (NOS). This combination is more effective in inhibiting NOS than either drug alone [31]; (2) 1H-[1,2,4]oxadiazolo[4,3-a]quinoxalin-1-one (ODQ, 10^−5^ M): a guanylate cyclase inhibitor [32]; (3) Verapamil (10^−5^ M): a L-type calcium channel blocker [33]; (4) Ryanodine (10^−5^ M) + 2-APB (3 × 10^−5^ M): inhibitors of the Ryanodine receptor (RyR) [34] and of the inositol triphosphate receptor (IP3R) [35], respectively; or (5) Paxilline (10^−5^ M): a blocker of large-conductance calcium-activated potassium channel (BKca) [25].

### 2.5. Drug and Solutions

HEPES-PSS was composed of the following compounds (in mmol/L): sodium chloride 141.8, potassium chloride 4.7, magnesium sulfate 1.7, calcium chloride 2.8, potassium phosphate 1.2, HEPES 10.0, EDTA 0.5, and dextrose 5.0. This solution was prepared using deionized water and titrated to a physiologic pH of 7.4 with sodium hydroxide (10 M).

The 80 mM high-potassium PSS depolarizing solution had an identical composition to the standard HEPES-PSS solution, except that NaCl was replaced with an equimolar concentration of KCl to maintain the osmolarity of the physiological solution. Chemicals were purchased from various sources, including Sigma-Aldrich (Merck KGaA, Darmstadt, Germany), Santa-Cruz Biotechnology, Inc. (Dallas, TX, USA), and Cayman Chemical Co (Ann Arbor, MI, USA), unless specified otherwise. All drugs used in the experiments were administered from daily prepared stock solutions, except for EVOO phenols, which were stored in small aliquots and frozen at −20 degrees Celsius.

### 2.6. Data Analysis and Statistics

The data are expressed as mean ± standard error of the mean (SEM), with n representing the number of experiments conducted. The vasodilatory effect of the EVOO phenols is expressed as the percentage of the maximal diameter, which was assessed in the presence of the relaxing solution. Potency is expressed as EC_50_, defined as the concentration of the dilator that elicited a 50% maximal response. Statistical analysis utilized the Area under the Curve (AUC) and was performed using an unpaired *t*-test. To determine significance, *p* < 0.05 was considered as significant.

## 3. Results

**Figure 1:**
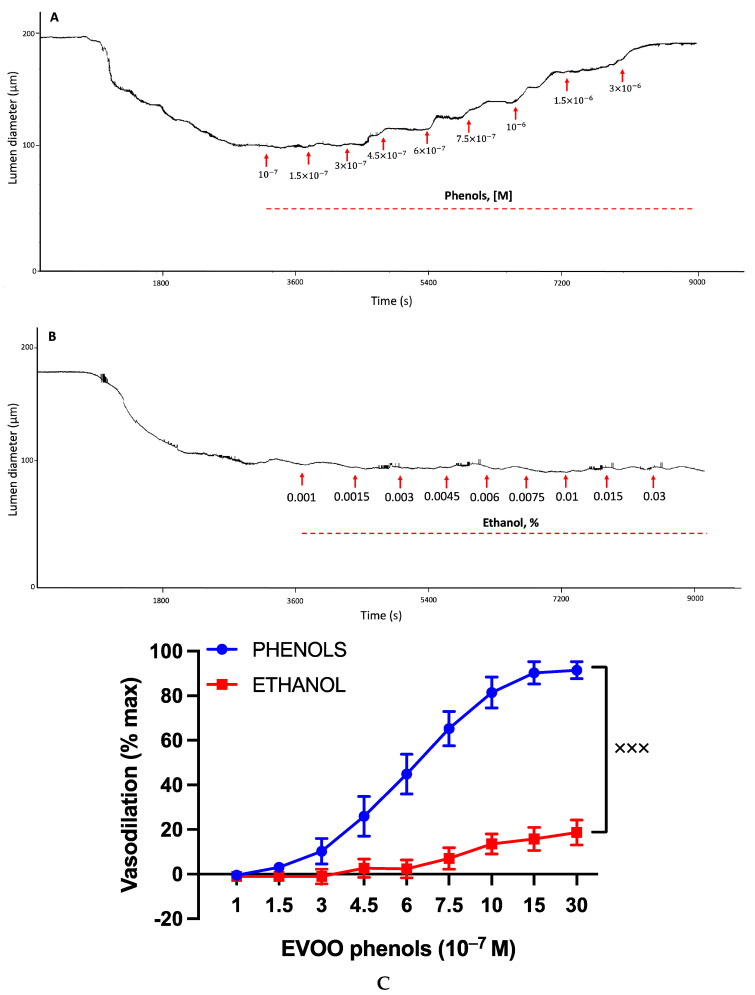
Effects of EVOO phenols and ethanol on uterine arteries. Uterine arteries, precontracted with phenylephrine, were tested with either EVOO phenols (Phenols, *n* = 15) or vehicle, ethanol (*n* = 6). Representative experiments are depicted in traces (**A**) and (**B**), respectively, while the cumulative results from multiple experiments are summarized in (**C**). Data are presented as mean ± SEM. Statistical analysis utilized the Area under the Curve (AUC) and was conducted using an unpaired *t*-test, ^xxx^ *p* < 0.001.

Figure 1 illustrates the effect of the EVOO phenols and ethanol on the UAs, and representative effects are depicted in traces Figure 1A and Figure 1B for EVOO phenols and ethanol, respectively. The collective results from multiple experiments are summarized in Figure 1C, which demonstrates the concentration-dependent vasodilation effect of the EVOO phenols on the UAs, reaching a maximum vasodilation value of 93.5 ± 6.9% and an EC_50_ of 6.0 × 10^−7^ M. In contrast, ethanol did not exhibit a significant vasodilation effect.

**Figure 2:**
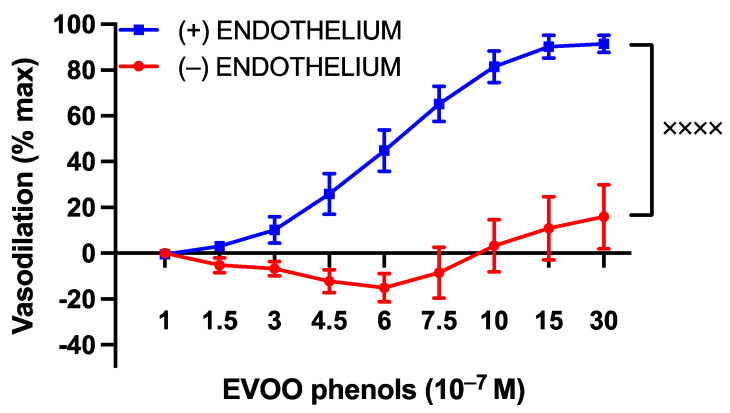
The influence of the endothelium on the vasodilatory effects of EVOO phenols in uterine arteries. EVOO phenols were tested on phenylephrine-precontracted uterine arteries with intact endothelium (+, *n* = 15) and without the endothelium (−, *n* = 5). The absence of the endothelium completely abolished the vasodilation effect of phenols. Data are presented as mean ± SEM, with n indicating the number of experiments. The Area under the curve (AUC) was considered for statistical analysis which was performed using unpaired *t*-test, ^xxxx^ *p* < 0.0001.

To investigate whether the vasodilatory effect of EVOO phenols was mediated by the endothelium, a series of experiments were performed on UAs that had been endothelium-denuded. As illustrated in Figure 2, the vasodilation induced by EVOO phenols was completely abolished in the denuded arteries compared to UAs with an intact endothelium.

**Figure 3:**
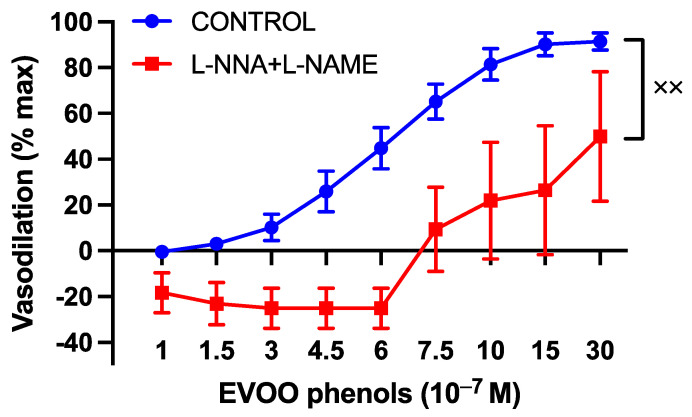
The involvement of nitric oxide in the vasodilatory effects of EVOO phenols on UAs. EVOO phenols were tested on phenylephrine-precontracted uterine arteries both in the absence (Control, *n* = 15) and in the presence of nitric oxide synthase enzyme inhibitors (L-NAME + L-NNA, *n* = 5). Data are presented as mean ± SEM, with n indicating the number of experiments conducted. The Area under the curve (AUC) was considered for statistical analysis which was performed using unpaired *t*-test, ^xx^ *p* < 0.01.

To explore the involvement of the potent endothelium-derived vasodilator nitric oxide (NO) in the vasodilatory effects of EVOO phenols on UAs, the EVOO phenols were assessed in the presence of NOS-specific inhibitors, L-NAME and L-NNA. The inhibitors were found to shift the phenols’ dose–response curve to the right, as shown in Figure 3.

**Figure 4:**
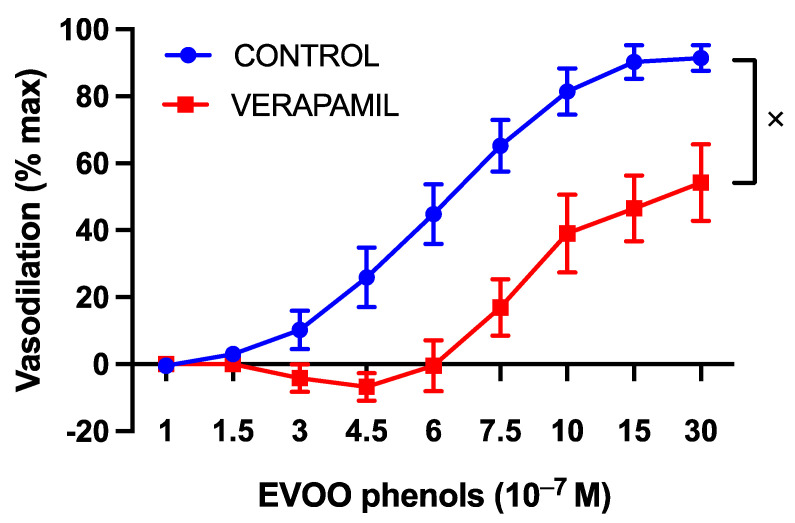
The involvement of L-type calcium channels in the vasodilatory effect of EVOO phenols on UAs. EVOO phenols were tested on phenylephrine-precontracted uterine arteries both in the absence (Control, *n* = 15) and in the presence of the L-type calcium channel blocker, Verapamil (*n* = 5). Data are presented as mean ± SEM, with n indicating the number of experiments conducted. The Area under the curve (AUC) was considered for statistical analysis which was performed using unpaired *t*-test, ^x^ *p* < 0.05.

Since endothelial nitric oxide synthase (eNOS) activation can occur in a calcium-dependent manner, the vasodilatory effect of the EVOO phenols was examined in the presence of calcium-signaling inhibitors. Verapamil, a specific L-type calcium channel blocker, induced a significant rightward shift in the dose–response curve of the phenols, as depicted in Figure 4.

**Figure 5:**
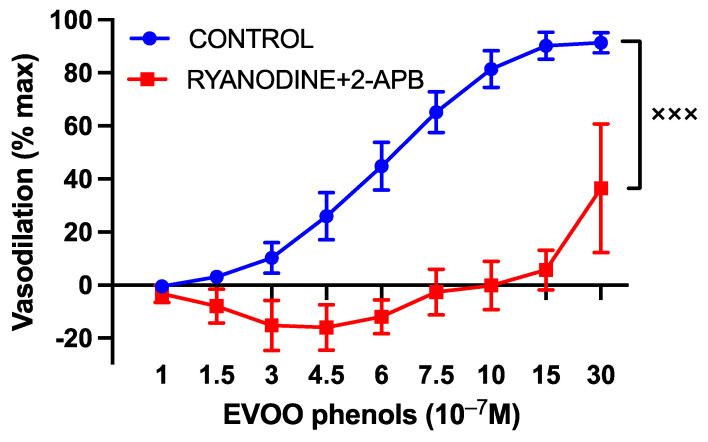
The involvement of endoplasmic reticulum calcium release in the vasodilatory effects of EVOO phenols on uterine arteries. EVOO phenols were tested on phenylephrine-precontracted uterine arteries both in the absence (control, *n* = 15) and in the presence of specific inhibitors of ryanodine (Ryanodine, 10^−5^ M, *n* = 5) and inositol triphosphate (2-APB, 3 × 10^−5^ M, *n* = 5). The inhibition of endoplasmic reticulum calcium release abolished phenols-induced vasodilation. Data are presented as mean ± SEM, with n indicating the number of experiments conducted. The Area under the curve (AUC) was considered for statistical analysis which was performed using unpaired *t*-test, ^xxx^ *p* < 0.001.

Moreover, the specific inhibitors of both ryanodine and IP3R receptors, Ryanodine (10 µM) and 2-APB (30 µM), respectively, which block the release of calcium from the endoplasmic reticulum, abolished phenols-induced vasodilation, as illustrated in Figure 5.

**Figure 6:**
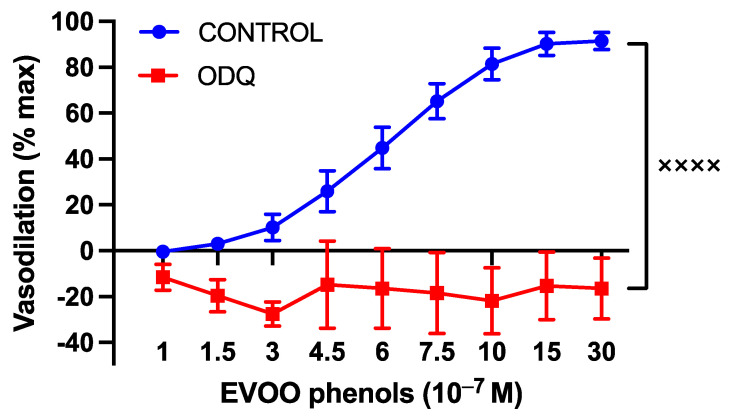
The involvement of cyclic guanosine monophosphate (cGMP) in the vasodilatory effects of EVOO phenols on uterine arteries. EVOO phenols were tested on phenylephrine-precontracted uterine arteries both in the absence (Control, *n* = 15) and in the presence of the cGMP inhibitor (ODQ, *n* = 5). Data are presented as mean ± SEM, with n indicating the number of experiments conducted. The Area under the curve (AUC) was considered for statistical analysis which was performed using unpaired *t*-test, ^xxxx^ *p* < 0.0001.

Moreover, to investigate NO-related mechanisms, a series of experiments were performed in the presence of ODQ (10^−5^ M), a specific guanylate cyclase inhibitor, and the BK_ca_ specific inhibitor, paxilline. The inhibition of cGMP production resulted in the abolition of phenols-induced vasodilation, as depicted in Figure 6.

**Figure 7:**
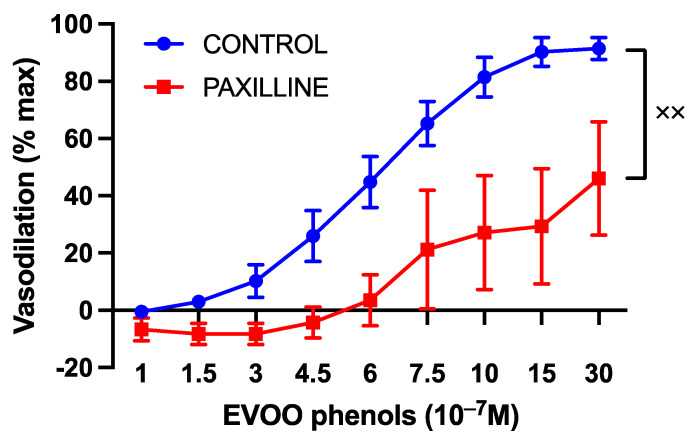
The impact of large-conductance calcium-activated potassium channels on the vasodilatory effects of EVOO phenols in uterine arteries. EVOO phenols were tested on phenylephrine-precontracted uterine arteries both in the absence (control, *n* = 15) and in the presence of the specific BK_ca_ channel inhibitor, Paxilline (*n* = 5). Data are presented as mean ± SEM, with n indicating the number of experiments conducted. The Area under the curve (AUC) was considered for statistical analysis which was performed using unpaired *t*-test, ^xx^ *p* < 0.01.

Additionally, the inhibition of BKca significantly attenuated EVOO phenols-induced vasodilation, as shown in Figure 7.

## 4. Discussion

In this study, we assessed the effect of EVOO phenols on UAs isolated from pregnant rats, revealing several key findings: (1) EVOO phenols vasodilated the UAs in a concentration-dependent manner, (2) the vasodilatory effect of the EVOO phenols was endothelium-dependent and mediated through the nitric oxide-cGMP-BKca pathways, and (3) EVOO phenol-induced vasodilation involved calcium signals via the activation of L-type calcium channels on the plasma membrane and ryanodine and IP3 receptors on the endoplasmic reticulum.

Our results align with previous studies demonstrating the vasodilatory effects of EVOO phenols on different types of vessels, such as rat aorta rings [36,37,38] and rat mesenteric arteries [25,26]. However, while the EVOO phenols act independently of the endothelium in the mesenteric arteries, our experiments highlighted an endothelium-dependent mechanism in UAs. Specifically, EVOO phenols initiated relaxation even at lower concentrations (starting at 0.3 µM) in endothelium-intact uterine arteries, whereas this effect was markedly inhibited in endothelium-denuded arteries, indicating an endothelium-dependent mechanism. This suggests that the mechanism of action of EVOO phenols varies depending on the vascular bed. Furthermore, it is plausible that phenols exert an enhanced action on endothelial cells during pregnancy due to increased endothelium-mediated vasodilation pathways [4], further supporting the endothelium-dependent pathway observed in UAs.

To further elucidate the endothelium-dependent mechanism underlying the vasorelaxant effects of EVOO phenols on UAs, a subsequent series of experiments was conducted. Since NO is a primary vasodilatory molecule synthesized by endothelial cells, EVOO phenols were tested in the presence of the nitric oxide synthase (eNOS) inhibitors L-NAME and L-NNA. The inhibition of NO production significantly reduced EVOO phenol-induced vasodilation, suggesting the involvement of NO in their mechanism of action. This finding is supported by previous studies demonstrating that exposure to polyphenolic compounds in HUVEC cells [37], as well as the in vivo treatment of rats [39], leads to increased NO production.

The significance of this vasodilation pattern is further emphasized by the heightened sensitivity to the NO-mediated vasodilation pathway during pregnancy [4]. However, the inhibition of NO did not entirely abolish EVOO-phenol-induced vasodilation. This partial inhibition could be attributed to the contribution from other endothelium-derived factors responsible for the endothelium-dependent hyperpolarization of vascular smooth muscle cells [40]. Additionally, the antioxidant properties of phenols may play a role by increasing the activity of NOS through the protection of tetrahydrobiopterin (a NOS cofactor) from oxidation. However, it should be noted that this study has a limitations, including the absence of assessments for cyclooxygenase (COX) inhibition and NOS activity, which could provide further insights into the mechanism underlying the vasodilatory effect of EVOO phenols. Therefore, additional studies are needed to elucidate these mechanisms comprehensively.

Based on our results, the vasodilation induced by EVOO phenols involved calcium signaling, as evidenced by the diminished vasodilatory response upon the inhibition of extracellular calcium influx and intracellular calcium mobilization from the endoplasmic reticulum. The ability of phenols to mobilize calcium ions from intracellular stores was also confirmed in cultured endothelial cells and appeared to be associated with increases in NO levels [41]. Additionally, D’Agostino et al. demonstrated the ability of phenols to activate phospholipase C (PLC), which, in turn, leads to calcium release from IP3-sensitive stores [26]. Consistent with these findings, we observed that the inhibition of IP3R and RYR, respectively, with 2-APB and ryanodine, decreased EVOO-phenol-induced vasodilation, underscoring the importance of intracellular calcium mobilization in their mechanism of action.

A hallmark of vasodilation pathways triggered by NO is the activation of soluble guanylate cyclase (sGC), which is responsible for producing cGMP. To investigate this, a series of experiments was conducted using ODQ, a specific cGMP inhibitor. ODQ completely abolished the vasodilation mediated by EVOO phenols, suggesting that cGMP plays a central role in their vasoactive action. cGMP can induce vasorelaxation through various pathways, including: (1) the inhibition of inositol-1,4,5-triphosphate generation; (2) enhanced cytosolic calcium extrusion; (3) the inhibition of calcium influx; (4) the activation of protein kinases; (5) the stimulation of membrane calcium ATPase; and (6) the opening of potassium channels [42]. Our study also suggests that BKca partly accounted for EVOO-phenol-induced vasodilation, as their inhibition reduced the vasodilatory effect. However, this partial reduction in vasodilation suggests the involvement of an additional mechanism mediated by cGMP, which requires further investigation for identification.

This finding holds significance for the potential use of EVOO phenols in treating pregnancies complicated by reduced uteroplacental blood flow and subsequent intrauterine growth restriction. The clinical implication of this study suggest that EVOO phenols could potentially be explored as a therapeutic option to improve uteroplacental perfusion and mitigate the risk of intrauterine growth restriction in affected pregnancies. Further research and clinical trials are warranted to validate these findings and assess the safety and efficacy of EVOO phenols in maternal–fetal medicine.

## 5. Conclusions

In conclusion, EVOO phenols demonstrate potent vasodilatory effects on uterine arteries. Their action is primarily endothelium-dependent, mediated mainly through the NO-cGMP pathway. To the best of our knowledge, this study represents the first evidence demonstrating the vasodilatory effects of EVOO phenols on rat UAs, offering new insights into their potential as novel therapeutic agents. Given their vasoactive properties, phenols could be valuable in improving compromised blood flow circulation in hypertensive pregnancies. Furthermore, EVOO phenols not only provide multiple health benefits, but, as natural compounds, also minimize the risk of side effects, highlighting their advantages in clinical use.

## Data Availability

Data will be provide by authors if needed.
